# Addressing Commonly Asked Questions in Urogynecology: Accuracy and Limitations of ChatGPT

**DOI:** 10.1007/s00192-025-06184-0

**Published:** 2025-06-18

**Authors:** Gregory Vurture, Nicole Jenkins, James Ross, Stephanie Sansone, Ellen Conner, Nina Jacobson, Scott Smilen, Jonathan Baum

**Affiliations:** 1 Division of Urogynecology, Department of Urology, Montefiore Medical Center—Albert Einstein College of Medicine, 1250 Waters Place, Tower Two, 9 th Floor, Bronx, NY 10460 USA; 2 Department of Obstetrics and Gynecology, Hackensack Meridian Health—Jersey Shore University Medical Center, Neptune City, NJ USA; 3 Division of Urogynecology, Department of Obstetrics and Gynecology, Hackensack Meridian Health—Jersey Shore University Medical Center, Neptune City, NJ USA

**Keywords:** Artificial intelligence, Large language model, Machine learning

## Abstract

**Introduction and Hypothesis:**

Existing literature suggests that large language models such as Chat Generative Pre-training Transformer (ChatGPT) might provide inaccurate and unreliable health care information. The literature regarding its performance in urogynecology is scarce. The aim of the present study is to assess ChatGPT’s ability to accurately answer commonly asked urogynecology patient questions.

**Methods:**

An expert panel of five board certified urogynecologists and two fellows developed ten commonly asked patient questions in a urogynecology office. Questions were phrased using diction and verbiage that a patient may use when asking a question over the internet. ChatGPT responses were evaluated using the Brief DISCERN (BD) tool, a validated scoring system for online health care information. Scores ≥ 16 are consistent with good-quality content. Responses were graded based on their accuracy and consistency with expert opinion and published guidelines.

**Results:**

The average score across all ten questions was 18.9 ± 2.7. Nine out of ten (90%) questions had a response that was determined to be of good quality (BD ≥ 16). The lowest scoring topic was “Pelvic Organ Prolapse” (mean BD = 14.0 ± 2.0). The highest scoring topic was “Interstitial Cystitis” (mean BD = 22.0 ± 0). ChatGPT provided no references for its responses.

**Conclusions:**

ChatGPT provided high-quality responses to 90% of the questions based on an expert panel’s review with the BD tool. Nonetheless, given the evolving nature of this technology, continued analysis is crucial before ChatGPT can be accepted as accurate and reliable.

**Supplementary Information:**

The online version contains supplementary material available at 10.1007/s00192-025-06184-0

## Introduction

Chat Generative Pre-training Transformer (ChatGPT) is an example of a Large Language Model (LLM) that has grown in popularity as a user-friendly interface where millions of users can find and generate answers to nearly an unlimited number of prompts. A recent version of ChatGPT, ChatGPT 3.5, is available to the general public through a free subscription. More advanced versions, such as ChatGPT-PLUS or GPT-4, have demonstrated an enhanced ability to understand and generate natural text in complex scenarios, but are accessible only via paid subscription.

Many editorial and opinion-based articles have been published within the general field of medicine, examining ChatGPT’s performance in clinical scenarios, reproducibility, and ability to pass entrance examinations for both foreign and domestic medical licensing qualifications [1–6]. When comparing performance among available LLMs, ChatGPT outperformed Google Bard and Bing in physiology-related vignettes, demonstrating higher potential for clinical and patient use [5].

In obstetrics and gynecology, Wan et al. cautioned against the use of ChatGPT, highlighting its poor performance in responding to pregnancy-related questions [7]. Meanwhile, Grünebaum et al. underscored ChatGPT’s potential to provide preliminary information on virtually any topic within the field [8]. In urology, several sources found that ChatGPT performed poorly, providing insufficient responses to urology health-related concerns [9–11]. Regarding subspecialties such as urogynecology, there are currently a limited number of publications evaluating the quality of ChatGPT’s responses to subspecialty-related scenarios, with none focusing specifically on ChatGPT’s responses to patient-based health questions.

The accuracy and validity of responses generated by LLMs must be evaluated before accepting its dissemination to the general public. At present, there is a lack of literature addressing their accuracy in the field of urogynecology; therefore, the aim of this study is to assess ChatGPT’s ability to accurately answer commonly asked questions in urogynecology based on published guidelines and through the use of a validated health care information assessment tool.

## Materials and Methods

### Formation of “Commonly Asked Questions”

An expert panel formulated a list of ten “commonly asked questions” in urogynecology. The expert panel consisted of 7 members: 5 American Board of Obstetrics and Gynecology (ABOG)-certified urogynecologists and 2 urogynecology fellows. Each of the ten questions addressed a different topic within urogynecology. The questions were written using verbiage and diction that a patient may use to ask their health care questions over the internet and agreed upon by the expert panel. Questions were not recalled from specific patient encounters but rather phrased based on the expert panel’s clinical experiences. Responses to the questions were scored using a validated tool based on their ability to be reliably and accurately answered based on the published guidelines of the American Urogynecologic Society (AUGS) and American Urological Association. Thus, ten questions were agreed upon that spanned different topics that may be addressed during a routine urogynecology visit.

### ChatGPT Utilization and Evaluation

ChatGPT version 3.5 was used to answer each question. At the time of the present study, GPT 3.5 was the only freely available version to the public and thus was favored for analysis compared with 4.0, which at the time, required a paid subscription. The responses were generated in November 2023. Each query was independently fed into ChatGPT in a separate instance. This was to avoid bias in its responses as the LLM can learn from prior context within the same instance, and therefore alter its responses. Answers were evaluated by the individual experts separately for appropriateness. Each response was assigned a score using the Brief DISCERN (BD) scoring system (Table 1), a validated health care information assessment questionnaire [12]. The BD tool consists of six scored questions that assess various qualities of the health care content including aim, whether the aim is addressed, relevance, sources, dates of sources, and bias [12]. Sensitivity and specificity of the BD tool was assessed by its original authors using a receiver operating characteristic (ROC) analysis. The tool was validated using 388 health care information websites and its validity was compared with the original DISCERN tool as its gold standard [15]. The lowest score a question can receive is 1 and the highest score can be 5. Thus, the lowest overall score possible is 6 and the highest possible score is 30. The ChatGPT responses were graded based on their accuracy and consistency with expert opinion and published guidelines. A score of ≥ 16 is consistent with good-quality content [12]. The panel was blinded from each individual expert’s BD scores to eliminate bias. At the conclusion of the study, individual scores and variation in scores were tracked.

**Table 1:** Brief Discern (BD) Scoring Tool

Question	No (score 1–2)	Partially (score 3–4)	Yes (score 5)
Are the aims clear?			
Does it achieve its aims?			
Is it relevant?			
Is it clear what sources of information were used to compile the publication (other than the author or producer)?			
Is it clear when the information used or reported in the publication was produced?			
Is it balanced and unbiased			
Total	/30		

### Statistical Analysis

Variation in the expert’s scoring of responses was analyzed using Fisher’s exact test. Statistical analysis was performed using JMP v.16.2 software (SAS Institute, Cary, NC, USA). All tests were two-sided with a significance level set at *p* < 0.05.

## Results

### Response Performance

**Fig. 1:**
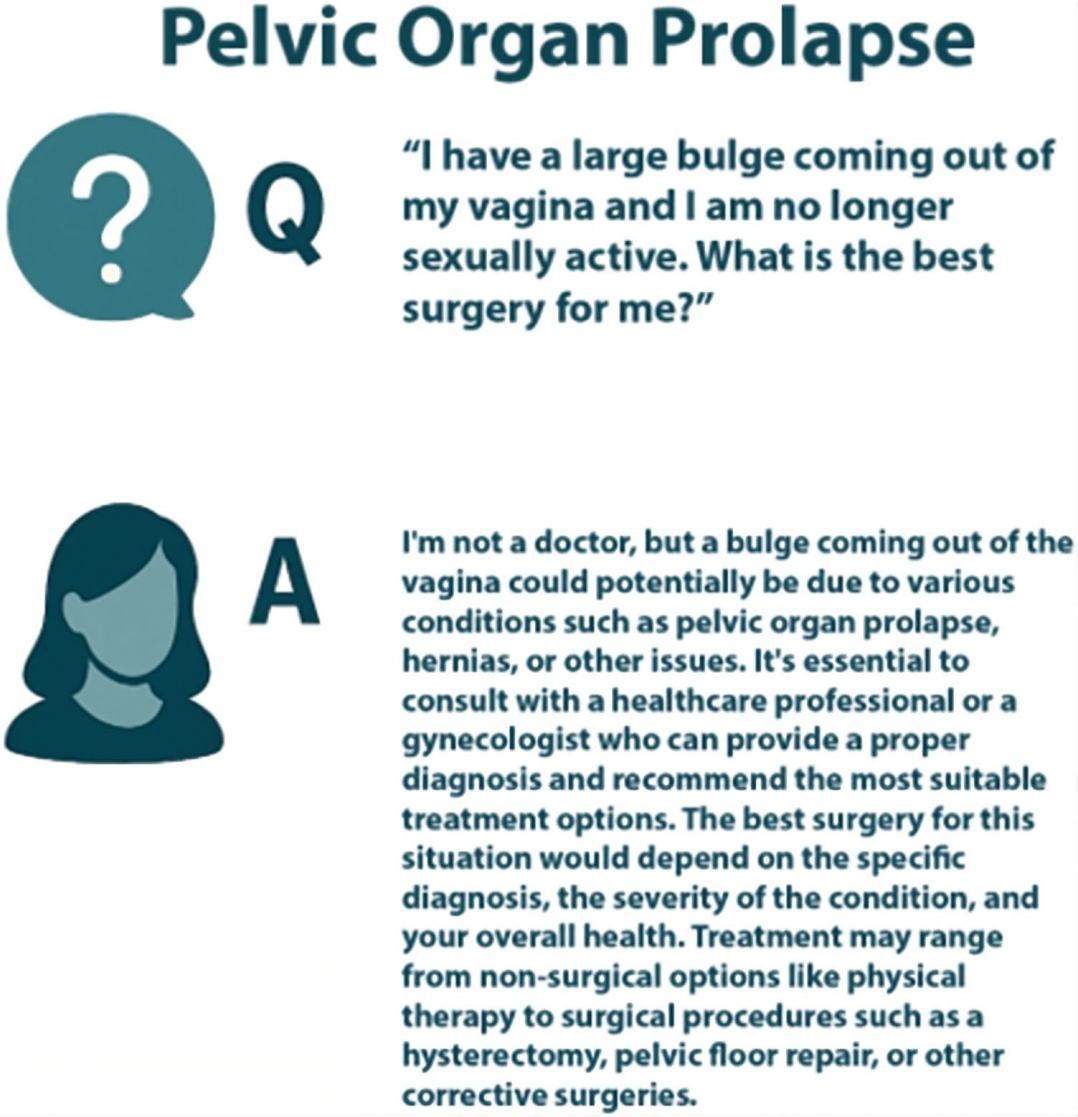
Pelvic organ prolapse question and answer

**Fig. 2:**
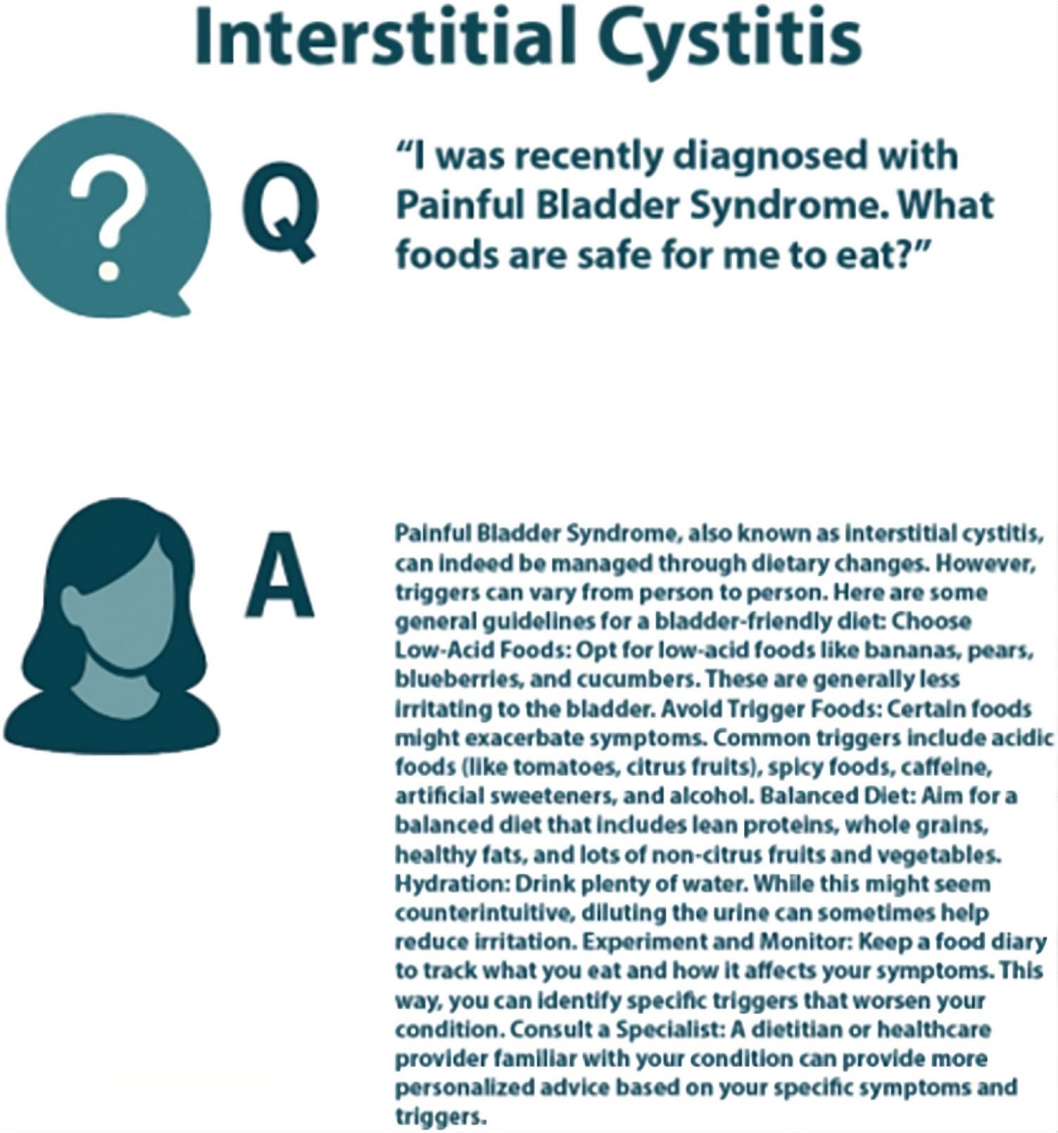
Interstitial cystitis question and answer

Nine out of ten (90%) questions had a response that was determined to be of good quality (BD ≥ 16). The average BD score across all ten questions was 18.9 ± 2.7 (Table 2). The lowest scoring topic was “Pelvic Organ Prolapse” (mean BD = 14.0 ± 2.0; Fig. 1). The highest scoring topic was “Interstitial Cystitis” (mean BD = 22.0 ± 0; Fig. 2). ChatGPT did not provide references across all its responses (Supplement 1).

**Table 2:** Evaluation of ChatGPT

Topic	Question	Mean BD score
Pelvic organ prolapse	“I have a large bulge coming out of my vagina and I am no longer sexually active. What is the best surgery for me?”	14.0 ± 2.0
Overactive bladder—medication options	“I leak urine when I have the urge to go but I want to avoid surgery. What medicine can I take?”	18.7 ± 2.2^a^
Interstitial cystitis	“I was recently diagnosed with painful bladder syndrome. What foods are safe for me to eat?”	22.0 ± 0*^a^
Stress incontinence treatment	“I leak when I cough, laugh, and sneeze. What’s my best treatment option?”	16.8 ± 2.3^a^
Pessary use	“My doctor prescribed me a pessary because my bladder is hanging low. How often should I clean my pessary?”	16.0 ± 3.2^a^
Midurethral slings—mesh complications	“My doctor and I discussed a midurethral sling but I saw that mesh in the vagina is bad. Is this safe?”	19.8 ± 1.6^a^
Hysterectomy route	“My doctor diagnosed me with pelvic organ prolapse. We talked about different types of hysterectomy. Which hysterectomy is the safest for me?”	19.5 ± 3.6^a^
Overactive bladder—medication safety	“I was prescribed medication for my overactive bladder. What side effects should I be concerned about?”	19.3 ± 1.6^a^
Obstetric anal sphincter injuries	“I had a tear to my rectum with my last vaginal delivery. Should I get a cesarean section for my next pregnancy?”	21.8 ± 0.4^a^
Anal incontinence	“I am leaking stool after my vaginal delivery. What are my treatment options? What surgery is recommended?”	21.5 ± 1.4^a^

^a^Indicates good quality content

### Variation Across Experts

There was minimal variation in responses across the experts. The greatest variation occurred with pessary use (16, IQR = 11–20). The least variation was with “Interstitial Cystitis” in which all experts unanimously gave a score of 22. When comparing expert response scores across all topics at the conclusion of the study, there were no statistically significant differences in blinded scores.

## Discussion

The present study demonstrated that ChatGPT provided high-quality and appropriate responses to 90% of patient-based urogynecological questions. ChatGPT appeared to be most thorough and accurate regarding its response for interstitial cystitis (mean BD = 22.0 ± 0) but did not perform as well regarding pelvic organ prolapse (mean BD = 14.0 ± 2.0). Although these results are promising, the current landscape of AI in urogynecology is limited at best.

A recent study evaluated ChatGPT’s performance in generating novel urogynecology research topics and found that 54% of the systematic review ideas were accurate [13]. Another study compared the ability of LLMs to identify AI generated urogynecological research abstracts compared with humans, and found that AI detection software was superior in this regard; however, performance varied greatly among LLMs, with Copyleaks only able to accurately detect 58.2% of ChatGPT-generated abstracts as AI written [14]. Another group evaluated ChatGPT 3.5, GPT-4, and Google Bard’s (now known as Gemini) performance on the AUGS self-assessment examination [15]. The authors found that GPT-4 performed best; however, none of the LLMs passed the examination with scores ranging from 42.7 to 61.6% [15]. Contrary to the above studies, a recent study demonstrated that ChatGPT performed well when written questions from International Urogynecological Association’s patient information leaflets regarding urogynecological procedures were fed into the LLM [16]. Most interestingly, performance of ChatGPT in its responses to the questions from the published patient leaflet improved when the study was repeated 3 months later, demonstrating the rapid nature by which these LLMs can improve depending on the resources publicly available from which it draws its conclusions [16].

In the landscape of general urology, a similar study to the present one was performed evaluating ChatGPT’s ability to answer urological guideline-based questions via evaluation with the BD score [9]. The authors of this study found that ChatGPT did not perform well, with only 54% of responses achieving a BD score greater than 16 [9]. The authors cautioned patient use of ChatGPT in the field of urology as it provided human-like responses that were inconsistent, unreliable, and inaccurate. Interestingly, this study was performed in February 2023, approximately 9 months prior to the present study. Therefore, it is very likely that the point in time in which health care questions are prompted into LLMs such as ChatGPT matters. The context of time is crucial to understanding its utility, accuracy, and most importantly, adaptability.

The rapidly evolving nature and easy accessibility to the public has allowed for many patients to turn to AI for answers to their medical questions. It is imperative that health care providers acknowledge where their patients are obtaining information. If a source is deemed unreliable, providers should provide resources for where to find accurate information. We cannot remain impartial to patients’ use of this technology. Therefore, it is crucial that information provided by LLMs is vetted to ensure that patients are receiving accurate evidence-based information before autonomously making health care decisions.

The present study has several important limitations. The patient questions were developed by an expert panel. These questions were crafted based on the authors’ experiences in the clinical setting using diction and verbiage that a patient may use. All questions were agreed upon unanimously by the panel. However, given that the questions were expert derived rather than patient derived, they may not have fully captured the linguistic and contextual nuances of an actual patient and therefore may have biased results. Additionally, the panel was from a single tertiary referral center, which may have biased judgments and not capture overall usage of AI. To combat bias, the BD tool [12] was utilized independently by each expert to rank the responses and the verbiage and diction of the questions were phrased to match patients as realistically as possible. Panelists were also blinded to each other’s scores to further limit bias. Also, the BD tool does not evaluate completeness of information and therefore scores may only reflect accurateness of what is present in ChatGPT’s response. Variation in responses was analyzed at the conclusion of the study. Although we found minimal variation across most of the topics, the topic identified to have the most variation was regarding pessaries. This may have stemmed from differing opinions regarding the response’s inability to provide a concise answer to timing of pessary changes with a rather thorough description of how to change and clean a pessary. We acknowledge these limitations and have included the patient-like questions and ChatGPT’s responses for interpretation by the reader (Supplement 1). We encourage readers to judge the responses themselves and develop their own queries for evaluation.

Another limitation is the time frame in which the study was performed. It is possible that if the present study were reproduced, ChatGPT may perform differently. LLMs such as ChatGPT generate their responses based on growing and expanding publicly available knowledge on the internet, information from third-party partners, and information that users or human trainers provide/generate [17]. Thus, as more individuals utilize and contribute to certain topics, the ability of LLMs to generate accurate responses may change with time. Additionally, we forwent using ChatGPT 4.0, despite its known better performance on topics within obstetrics and gynecology [17], as at the time that the present study was performed, it was not freely available to the public. This may have resulted in selection bias. Regardless, the present study provides a snapshot in time of ChatGPT’s performance across various topics in urogynecology. Given the ever-evolving nature of LLMs such as ChatGPT, past performance of AI is absolutely crucial to the implications, understanding, and evaluation of AI in the present and for the future. Therefore, continued research and analysis of ChatGPT and other LLMs are necessary and crucial before it may be adopted as an accurate and reliable source for urogynecological information by patients.

## Supplementary Information

Below is the link to the electronic supplementary material.

Supplement 1 ChatGPT questions and answers (DOCX 18 KB)

## Data Availability

All data supporting the findings of this study are available within the paper and its Supplementary Information. Supplement 1 includes both the ChatGPT prompts and responses so that the reader may interpret the responses themselves and develop their own queries for evaluation
