# Supplementary material for: Addressing Commonly Asked Questions in Urogynecology: Accuracy and Limitations of ChatGPT
Source: Int Urogynecol J. 2025 Jun 18;36(11):2249–54. doi: 10.1007/s00192-025-06184-0 (PMC12681482; doi:10.1007/s00192-025-06184-0)
Supplement: Supplementary file 1 — Supplement 1 ChatGPT questions and answers (DOCX 18 KB) [file 192_2025_6184_MOESM1_ESM.docx]

**Supplement - ChatGPT Questions and Answers**

| **Topic** | **Question** | **ChatGPT 3.5 Answer - Nov 2023** |
| --- | --- | --- |
| **Pelvic Organ Prolapse** | “I have a large bulge coming out of my vagina and I am no longer sexually active. What is the best surgery for me?” | I'm not a doctor, but a bulge coming out of the vagina could potentially be due to various conditions such as pelvic organ prolapse, hernias, or other issues. It's essential to consult with a healthcare professional or a gynecologist who can provide a proper diagnosis and recommend the most suitable treatment options. The best surgery for this situation would depend on the specific diagnosis, the severity of the condition, and your overall health. Treatment may range from non-surgical options like physical therapy to surgical procedures such as a hysterectomy, pelvic floor repair, or other corrective surgeries. |
| **Overactive Bladder - Medication Options** | “I leak urine when I have the urge to go but I want to avoid surgery. What medicine can I take?” | I'm not a doctor, but I can provide some general information. Urinary incontinence can have various causes, and the appropriate treatment depends on the underlying reason for the issue. It's crucial to consult a healthcare professional for proper diagnosis and treatment advice tailored to your specific situation. Medications might be prescribed for certain types of urinary incontinence, such as: Overactive Bladder (OAB): Medications like anticholinergics or beta-3 agonists can help relax the bladder muscles, reducing the urgency and frequency of urination. Stress Incontinence: There might not be specific medications for this type, but pelvic floor exercises (Kegels) could be recommended to strengthen the muscles supporting the bladder. Overflow Incontinence: Medications to reduce urinary retention might be prescribed if the issue stems from an obstruction or incomplete bladder emptying. However, if your incontinence is caused by structural problems or other issues that medications can't resolve, surgery might be the most effective option. Surgery can often provide long-term relief in cases where medications or other conservative treatments aren't effective. Remember, it's essential to discuss your symptoms thoroughly with a healthcare professional to determine the best course of action based on your specific situation and medical history. |
| **Interstitial Cystitis** | “I was recently diagnosed with Painful Bladder Syndrome. What foods are safe for me to eat?” | Painful Bladder Syndrome, also known as interstitial cystitis, can indeed be managed through dietary changes. However, triggers can vary from person to person. Here are some general guidelines for a bladder-friendly diet: Choose Low-Acid Foods: Opt for low-acid foods like bananas, pears, blueberries, and cucumbers. These are generally less irritating to the bladder. Avoid Trigger Foods: Certain foods might exacerbate symptoms. Common triggers include acidic foods (like tomatoes, citrus fruits), spicy foods, caffeine, artificial sweeteners, and alcohol. Balanced Diet: Aim for a balanced diet that includes lean proteins, whole grains, healthy fats, and lots of non-citrus fruits and vegetables. Hydration: Drink plenty of water. While this might seem counterintuitive, diluting the urine can sometimes help reduce irritation. Experiment and Monitor: Keep a food diary to track what you eat and how it affects your symptoms. This way, you can identify specific triggers that worsen your condition. Consult a Specialist: A dietitian or healthcare provider familiar with your condition can provide more personalized advice based on your specific symptoms and triggers. |
| **Stress Incontinence Treatment** | “I leak when I cough, laugh, and sneeze. What’s my best treatment option?” | It sounds like you might be experiencing urinary incontinence, which can happen when there's a weakness in the muscles that control the release of urine. The best treatment options depend on the cause and severity of your symptoms. Here are some possibilities: Pelvic Floor Exercises: Known as Kegel exercises, these can strengthen the muscles that control urine flow and help reduce leakage. Lifestyle Changes: Sometimes, dietary adjustments (like reducing caffeine intake) or maintaining a healthy weight can alleviate symptoms. Behavioral Techniques: Timed voiding or bladder training can help you gradually increase the time between bathroom visits. Medications: There are medications that can help relax the bladder or reduce urinary urgency. Medical Devices: For some, devices like pessaries (for women) or urethral inserts can provide support to reduce leakage. Surgery: In severe cases, surgery might be considered to provide additional support to the bladder or urethra. It's important to discuss your symptoms with a healthcare professional to determine the cause and severity of your urinary incontinence. They can then recommend the best treatment options tailored to your specific situation. |
| **Pessary Use** | “My doctor prescribed me a pessary because my bladder is hanging low. How often should I clean my pessary?” | Cleaning your pessary is essential to maintain hygiene and prevent infection. The frequency of cleaning can depend on the type of pessary and your doctor's recommendations. However, a general guideline is to clean the pessary regularly: Daily Rinsing: Rinse your pessary daily with mild soap and water. Ensure it's thoroughly cleaned and rinsed to remove any soap residue. Proper Drying: Allow the pessary to air dry completely before using it again. Moisture can promote bacterial growth, so ensuring it's dry is crucial.Always follow your doctor's instructions regarding the care and cleaning of your pessary. If you experience any discomfort, irritation, or notice an unusual odor, contact your healthcare provider promptly. Additionally, regular follow-up appointments with your doctor are important to ensure the pessary fits properly and continues to effectively support your condition. They can also provide guidance on proper care and usage. |
| **Midurethral Slings - Mesh Complications** | “My doctor and I discussed a midurethral sling but I saw that mesh in the vagina is bad. Is this safe?” | It's understandable to have concerns about mesh implants, given the history of issues reported with some types of vaginal mesh used in surgeries like pelvic organ prolapse repair or stress urinary incontinence. Mesh slings used in midurethral sling procedures for stress urinary incontinence have been associated with complications, but they can also be effective for many women with fewer complications. It's important to have an open conversation with your doctor about the specific type of sling they recommend, the risks involved, and the likelihood of benefits. Advances have been made in mesh technology, and newer materials and surgical techniques might mitigate some of the risks associated with older mesh implants. Ask your doctor about the specifics: the type of mesh they intend to use, the procedure's success rates, potential complications, and alternative treatments available. Staying informed and discussing your concerns openly with your healthcare provider will help you make a well-informed decision about your treatment options. |
| **Hysterectomy Route** | “My doctor diagnosed me with pelvic organ prolapse. We talked about different types of hysterectomy. Which hysterectomy is the safest for me?” | The safety and suitability of each type of hysterectomy depend on individual circumstances. Factors like the size of the uterus, your general health, previous surgeries, and the expertise of your surgeon play crucial roles in determining which approach is safest and most effective for you. It's essential to have an in-depth discussion with your doctor/surgeon about the benefits, risks, and expected outcomes of each type of hysterectomy concerning your specific condition and health status. |
| **Overactive Bladder - Medication Safety** | “I was prescribed medication for my Overactive Bladder. What side effects should I be concerned about?” | It's crucial to stay informed about potential side effects when taking medication for an overactive bladder. Common side effects might include: Dry mouth: This is a frequent side effect due to the medication affecting saliva production. Constipation: Some medications can lead to difficulty passing stool. Blurred vision: In rare cases, certain medications might affect eyesight temporarily. Dizziness or drowsiness: Some people may feel lightheaded or sleepy after taking these medications. Difficulty urinating: Paradoxically, in some instances, these medications might cause difficulty in initiating urination. Allergic reactions: Though rare, allergic reactions like rashes, itching, or swelling may occur. Always consult your healthcare provider if you notice any concerning symptoms or experiences that seem unusual after starting a new medication. They can provide guidance or adjust your treatment plan accordingly. |
| **Obstetric Anal Sphincter Injuries (OASIS)** | “I had a tear to my rectum with my last vaginal delivery. Should I get a cesarean section for my next pregnancy?” | I can understand your concern. A tear to the rectum during vaginal delivery, known as a third or fourth-degree tear, can be a distressing experience. It's a decision that should be made in consultation with your obstetrician or healthcare provider. They can assess your individual situation, discuss the risks and benefits of both vaginal birth after a tear and cesarean section, and help you make an informed choice based on your health and circumstances. Factors they might consider include the severity of the tear, any ongoing symptoms or issues, the likelihood of a similar tear happening again, and your preferences for future deliveries. Cesarean section does reduce the risk of recurrent severe tears, but it also carries its own set of risks and considerations. Ultimately, your healthcare provider will work with you to determine the safest option for you and your baby in your next pregnancy. It's essential to have an open discussion with them to address your concerns and make an informed decision that aligns with your health goals. |
| **Anal Incontinence** | “I am leaking stool after my vaginal delivery. What are my treatment options? What surgery is recommended?” | I'm not a doctor, but I can provide some general information. After a vaginal delivery, some women may experience issues like stool leakage, which could be due to damage to the muscles and nerves in the pelvic floor. The condition you're describing is often referred to as fecal incontinence. Treatment options can vary based on the severity and underlying cause. Here are some possible approaches: Conservative Treatments: Pelvic floor exercises (Kegel exercises) can help strengthen the muscles around the anus and vagina. Dietary changes, like increasing fiber intake, can regulate bowel movements. Medications may be prescribed to manage diarrhea or improve bowel movements. Biofeedback Therapy: This technique helps you learn how to control and strengthen your pelvic floor muscles. Surgery: In severe cases where conservative treatments don't work, surgery might be considered. Surgical options include repairing damaged muscles or nerves, placing a device to support the anus (sphincteroplasty), or other procedures based on the specific underlying issue. The type of surgery recommended would depend on various factors such as the cause and severity of the problem. It's crucial to consult with a healthcare professional, typically a colorectal surgeon or a specialist in pelvic floor disorders, who can assess your condition and suggest the most appropriate treatment plan for you. Remember, discussing these concerns openly with your healthcare provider is crucial for receiving personalized guidance and appropriate care. |
